# Lung ultrasound in a tertiary intensive care unit population: a diagnostic accuracy study

**DOI:** 10.1186/s13054-021-03759-3

**Published:** 2021-09-17

**Authors:** Jasper M. Smit, Mark E. Haaksma, Michiel H. Winkler, Micah L. A. Heldeweg, Luca Arts, Erik J. Lust, Paul W. G. Elbers, Lilian J. Meijboom, Armand R. J. Girbes, Leo M. A. Heunks, Pieter R. Tuinman

**Affiliations:** 1 Department of Intensive Care Medicine, Research VUmc Intensive Care (REVIVE) and Amsterdam Cardiovascular Sciences (ACS), Amsterdam UMC, Location VU University Medical Center, de Boelelaan 11171007MB, Postbox 7505, Amsterdam, The Netherlands; 2 Amsterdam Leiden Intensive Care Focused Echography (ALIFE), Amsterdam, The Netherlands; 3 Department of Radiology and Nuclear Medicine, Amsterdam Cardiovascular Sciences (ACS), Amsterdam UMC, Vrije Universiteit Amsterdam, de Boelelaan 1117, Amsterdam, The Netherlands

**Keywords:** Lung ultrasound, Thoracic computed tomography, Chest CT, Diagnostic accuracy, Acute respiratory failure

## Abstract

**Background:**

Evidence from previous studies comparing lung ultrasound to thoracic computed tomography (CT) in intensive care unit (ICU) patients is limited due to multiple methodologic weaknesses. While addressing methodologic weaknesses of previous studies, the primary aim of this study is to investigate the diagnostic accuracy of lung ultrasound in a tertiary ICU population.

**Methods:**

This is a single-center, prospective diagnostic accuracy study conducted at a tertiary ICU in the Netherlands. Critically ill patients undergoing thoracic CT for any clinical indication were included. Patients were excluded if time between the index and reference test was over eight hours. Index test and reference test consisted of 6-zone lung ultrasound and thoracic CT, respectively. Hemithoraces were classified by the index and reference test as follows: consolidation, interstitial syndrome, pneumothorax and pleural effusion. Sensitivity, specificity, positive and negative likelihood ratio were estimated.

**Results:**

In total, 87 patients were included of which eight exceeded the time limit and were subsequently excluded. In total, there were 147 respiratory conditions in 79 patients. The estimated sensitivity and specificity to detect consolidation were 0.76 (95%CI: 0.68 to 0.82) and 0.92 (0.87 to 0.96), respectively. For interstitial syndrome they were 0.60 (95%CI: 0.48 to 0.71) and 0.69 (95%CI: 0.58 to 0.79). For pneumothorax they were 0.59 (95%CI: 0.33 to 0.82) and 0.97 (95%CI: 0.93 to 0.99). For pleural effusion they were 0.85 (95%CI: 0.77 to 0.91) and 0.77 (95%CI: 0.62 to 0.88).

**Conclusions:**

In conclusion, lung ultrasound is an adequate diagnostic modality in a tertiary ICU population to detect consolidations, interstitial syndrome, pneumothorax and pleural effusion. Moreover, one should be careful not to interpret lung ultrasound results in deterministic fashion as multiple respiratory conditions can be present in one patient.

*Trial registration* This study was retrospectively registered at Netherlands Trial Register on March 17, 2021, with registration number NL9344.

**Supplementary Information:**

The online version contains supplementary material available at 10.1186/s13054-021-03759-3.

## Background

Many patients admitted to the intensive care unit (ICU) meet criteria for acute respiratory failure [1]. Common causes for respiratory failure in these patients include cardiogenic pulmonary edema (CPE), acute respiratory distress syndrome (ARDS), atelectasis and pneumonia [2]. To date, to detect these conditions chest X-ray and thoracic computed tomography (CT) are still the most common diagnostic modalities. However, recent years have seen an increase in the use of lung ultrasound [3, 4].

Above-mentioned respiratory conditions are associated with increased attenuation on thoracic CT, which can be divided into ground-glass opacity and consolidations. Consolidations are, for example, often caused by pneumonia or atelectasis, in contrast to CPE, which is more associated with ground-glass opacities [5]. Lung ultrasound can accurately detect these thoracic CT findings and provide a less costly, ionizing radiation-free diagnostic modality without the risk of transportation [6]. Previous studies have already shown that so-called B line artifacts on lung ultrasound are associated with an increased amount of pulmonary edema and thus correlate with linear and ground-glass opacities on thoracic CT [7, 8]. Moreover, consolidations are readily detected by lung ultrasound because they are usually the result of replacement of air by fluid or cells and are, therefore, easily traversed by ultrasound waves [3, 9].

Previous studies relating lung ultrasound to thoracic CT findings in ICU patients, however, were hampered by multiple methodologic limitations. Most studies excluded patients with multiple diagnoses, were inadequately blinded, or did not include anterior consolidations and/or pneumothorax [8, 10–15]. Subsequently, a study that is appropriately blinded and investigates the full diagnostic extent of lung ultrasound in an ICU population was needed [7, 16].

As follows, the primary aim of this study was to evaluate the diagnostic accuracy of a 6-zone lung ultrasound protocol in ICU patients, with thoracic CT as reference standard, using adequate blinding and including patients with multiple respiratory conditions. Secondary aims were: 1. To evaluate the diagnostic accuracy of an extended 12-zone lung ultrasound protocol and 2. To correlate lung ultrasound patterns with respiratory conditions in a tertiary ICU population, while taking into account that one patient could have multiple lung ultrasound abnormalities, but also be affected by multiple respiratory conditions.

## Methods

### Study design

This was a single-center, prospective, observational diagnostic accuracy study conducted at a tertiary intensive care unit (ICU) of Amsterdam UMC, location VU university medical center, in Amsterdam, the Netherlands. This study was approved by the local medical ethics review committee (METc VUmc, ID: 2016.002) and the necessity for informed consent was waived. Patients were enrolled between January 2016 and January 2019. ‘Standards for Reporting Diagnostic accuracy studies’ (STARD)-guidelines were followed (Additional file 1) [17].

### Study population

Adult patients (≥ 18 years) admitted to the ICU who underwent thoracic CT for any clinical indication were included. Population consisted of mechanically and nonmechanically ventilated patients. Patients were excluded if thoracic CT and lung ultrasound examination were more than 8 h apart. Study population was composed of a prospective random sample. All lung ultrasound operators (n = 17) were blinded for any CT findings. They were trained according to a Dutch Intensive Care ultrasound course, which entailed a minimum of 40 supervised examinations by an experienced ultrasound operator [4]. The following patient characteristics were collected from the electronic patient record: sex, age, body mass index, past medical history, sequential organ failure assessment (SOFA) score at day of CT-scan, reason for ICU admission, indication for CT-scan and respiratory conditions.

### Index test

**Fig. 1:**
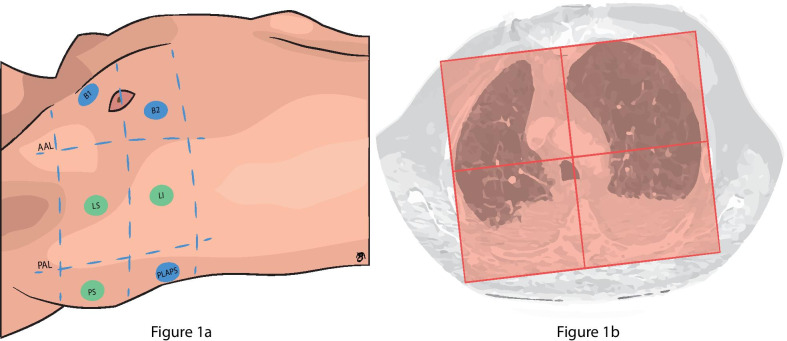
**a** ‘Bedside Lung Ultrasound in Emergency’ (BLUE) and 12-zone lung ultrasound protocol. The blue points represent BLUE-points of the lung ultrasound protocol, they are identified as follows: two hands are placed consecutively just below the clavicle, excluding the thumbs. The upper BLUE-point is at the middle of the upper hand, whereas the Lower BLUE-point is at the middle of the lower palm. The posterolateral alveolar and/or pleural syndrome (PLAPS)-point is just below the posterior axillary line at the height of the diaphragm. At the PLAPS-point the probe was moved posteriorly in order to obtain as much information as possible about the posterior lung region. The green points are scanned additionally in the 12-zone lung ultrasound protocol. AAL: anterior axillary line; PAL: posterior axillary line; B1: BLUE-1; B2: BLUE-2; LS: lateral superior; LI: lateral inferior; PS: posterior superior; PLAPS: posterolateral alveolar and/or pleural syndrome. **b** A schematic image of how thoracic CT was divided into two hemithoraces and an anterior and posterior region. Two lines were drawn tangent to the anterior and posterior border of both lungs and at the height of the sternum anteriorly and spinous process posteriorly a line perpendicular to the tangent lines was drawn to divide the thoracic cavity into a right and left side. Both lungs were then divided into an anterior and posterior half by drawing a line perpendicular from the middle of the line between the sternum and spinous process to the lateral border of the lung. Each hemithorax was evaluated for anterior consolidations, posterior consolidations, interstitial syndrome, pneumothorax or pleural effusion

Index test consisted of a 6-zone lung ultrasound examination according to the Bedside Lung Ultrasound in Emergency (BLUE)-protocol [7]. This protocol entailed scanning three standardized points per hemithorax [16]. The upper and lower BLUE-points were used to evaluate the anterior lung region, and the posterolateral alveolar and/or pleural syndrome (PLAPS)-point was used to evaluate the posterior lung region (Fig. 1a). Ultrasound evaluations were performed with the Philips CX50 (Koninklijke Philips NV, The Netherlands) or SonoSite Edge II (Fujifilm SonoSite Inc., USA). A linear high-frequency transducer was used to evaluate the anterior lung region. For the PLAPS-point a low-frequency, convex, phased array transducer was used. During ultrasound evaluation with the linear transducer, artifact eliminating software was turned off.

At the BLUE-points the pleura was identified and it was noted whether lungsliding was present [18]. Furthermore, artifacts and real images below the pleura were assessed as described previously [7]. In case of predominant A-lines, an A-profile was designated to the assessed hemithorax. A B-profile was designated to the assessed hemithorax in case of three or more B-lines at the upper or lower BLUE-point. A C-profile was designated to the assessed hemithorax in case of a subpleural hypoechoic image restricted by an irregular border (shred sign) and overruled B-profile if present [19].

At the PLAPS-point the presence of pleural effusion or consolidation was assessed. Pleural effusion was diagnosed in case of an anechoic area between parietal and visceral pleura. Consolidation was diagnosed in case of a subpleural echo-poor image restricted by an irregular border or a tissue-like pattern of the lung on ultrasound—similar to abdominal parenchyma—corresponding to complete loss of aeration [19].

Each hemithorax was classified according to the following lung patterns:

1. Anterior consolidation: C-profile or B-profile without lungsliding (B’-profile) at the upper or lower BLUE-point.
2. Posterior consolidation: hypoechoic image restricted by an irregular border or a tissue-like pattern at the PLAPS-point.
3. Interstitial syndrome: B-profile with lungsliding at upper or lower BLUE-point. This classification requires the absence of an anterior consolidation.
4. Pneumothorax: A-profile without lungsliding at upper or lower BLUE-point.
5. Pleural effusion: anechoic or hypoechoic area between parietal and visceral pleura at any point.

To potentially increase the sensitivity of lung ultrasound, a more elaborate 12-zone protocol was used in a subgroup of patients. In this group, besides the BLUE-points and PLAPS-point, the anterior axillary line and posterior axillary line were divided into a superior and inferior half (Fig. 1a). The two points scanned below the anterior axillary line were regarded as anterior lung regions and the two points scanned below the posterior axillary line as posterior lung regions. These points were evaluated in identical fashion as described above.

### Reference test

Reference test consisted of thoracic CT. Patients were scanned in the emergency room or radiology department with a Discovery CT750 HD CT scanner (GE Healthcare, USA) or SOMATOM Drive Dual Source CT scanner (Siemens Healthineers, Germany), respectively. The following CT variables were used: tube voltage 70 to 140 kV, rotation time 0.27 to 2 s, collimation 64 × 0.5 mm.

CT images were assessed and reported by a radiologist blinded for the results of lung ultrasound examination. All images were evaluated using a mediastinal window and a lung window setting. Mediastinal window width was 360 Hounsfield units (HU) and level was 20 HU. Lung window width and level were 1600 HU and -600 HU, respectively.

An independent researcher, blinded for the lung ultrasound examinations, divided each hemithorax into an anterior and posterior half in the axial plane throughout the entire chest (Fig. 1b). Subsequently, lung findings reported by the radiologist were then adjudicated to their respective location (anterior or posterior). Each hemithorax on thoracic CT was classified according to the following lung patterns [5, 20]:

1. Anterior consolidation: increase in pulmonary parenchymal attenuation that obscures margins of the vessels and airways.
2. Posterior consolidation: increase in pulmonary parenchymal attenuation as described above.
3. Interstitial syndrome: ground-glass or linear opacities. Ground-glass opacities were defined as hazy increase in pulmonary parenchymal attenuation with preservation vascular and bronchial margins. Linear opacities were defined as the thickening of the peribronchovascular, subpleural or interlobular septa. This classification requires the absence of an anterior consolidation.
4. Pneumothorax: accumulation of air between the visceral and parietal pleura.
5. Pleural effusion: accumulation of fluid between the visceral and parietal pleura.

### Outcomes

Primary outcome was the diagnostic accuracy of lung ultrasound to detect consolidation (anterior or posterior consolidation), interstitial syndrome, pneumothorax and pleural effusion as compared to thoracic CT. Accuracy outcome parameters were sensitivity, specificity, positive likelihood ratio and negative likelihood ratio. A ‘true positive’ result was defined as ultrasound suggested the presence of a specific lung pattern confirmed by thoracic CT. A ‘true negative’ result was defined as ultrasound suggested the absence of a specific lung pattern confirmed by thoracic CT.

Two secondary outcomes were determined. First, the diagnostic accuracy of an extended, 12-zone lung ultrasound protocol (Fig. 1a). Accuracy outcome parameters were sensitivity, specificity, positive likelihood ratio and negative likelihood ratio.

Second, lung ultrasound patterns were correlated with respiratory conditions. Included conditions were atelectasis, pneumonia, cardiogenic pulmonary edema, acute respiratory distress syndrome (ARDS), pneumothorax, lung contusion and lung infarction. If a patient had multiple conditions, for example pneumothorax and lung contusion, both were included in the analyses. The presence of respiratory conditions was based on the radiology report of the thoracic CT images and clinical interpretation of the report by the treating physician. The lung ultrasound operator was blinded for the results of the radiologist and treating physician and vice versa.

### Statistical Analysis

Sample size calculation was based on an average estimated prevalence of lung abnormalities on CT of 0.57, an average sensitivity and specificity of 0.92 and 0.91, respectively, and a margin of error of 0.07. [8, 21] With these parameters the estimated required sample size was 150. Data were expressed as mean (± SD) or as median [IQR] when appropriate. To assess distribution, histograms and Q-Q plots were evaluated. Categorical variables were expressed as numbers and percentages. Sensitivity, specificity, positive and negative likelihood ratio were estimated with their respective 95% confidence interval [22]. Confidence intervals for sensitivity and specificity were estimated using a robust sandwich variance estimator to account for clustering of two hemithoraces per patient. All analyses were performed in R (RStudio, USA).

## Results

For primary analysis, 87 patients were included in two distinct time periods: from January 2016 until July 2016 (24 weeks) and September 2017 to January 2019 (70 weeks). In eight patients thoracic CT and lung ultrasound were not performed within the timeframe of eight hours and were subsequently excluded. There was no missing data, except in five patients one of the two PLAPS-points could not be accurately visualized due to thoracic drains or surgical dressings and these were excluded from consolidation and pleural effusion analyses. Baseline characteristics are described in Table 1. The results are depicted in Table 2. For secondary analysis (patients who received a 12-zone lung ultrasound examination) 18 patients were included. The results are depicted in Table 3. The results per lung pattern are described below.

**Table 1:** Baseline characteristics

Patient characteristics	Overall (N = 79) N (%), mean (±SD), median [IQR]
Gender (%)	
Male	53 (67.1)
Female	26 (32.9)
Age, yr	60.4 (± 15.7)
BMI, kg/m^2^	25.3 [22.6, 28.7]
Reason for admission (%)	
Cardiovascular	15 (19.0)
Gastrointestinal	4 (5.1)
Hematological	13 (16.5)
Pulmonary	35 (44.3)
Sepsis	3 (3.8)
Trauma	9 (11.4)
CT indication (%)	
Medical	40 (50.6)
Surgical	21 (26.6)
Cardiac and respiratory arrest	9 (11.4)
Trauma	9 (11.4)
Mechanical ventilation (%)	
Yes	62 (78.5)
No	17 (21.5)
Number of respiratory conditions (%)	
0	1 (1.3)
1	24 (30.4)
2	39 (49.4)
3	15 (19.0)
Time between LU and CT	2.5 [1.25, 3.0]
PEEP, cmH2O	10.0 [8.0, 12.0]
P/F ratio	159.2 [116.8, 209.1]
CRP, mg/L	115.5 [26.5, 281.8]
Leucocytes, × 10^9^/L	11.5 [6.8, 16.3]
SOFA score	8.0 [6.0, 12.0]

Table depicting baseline characteristics. BMI: body mass index; CT: computed tomography; LU: lung ultrasound; PEEP: positive end expiratory pressure; P/F ratio: PaO2 / FiO2 ratio; CRP: c-reactive protein; SOFA: sequential organ failure assessment; SD: standard deviation; IQR: interquartile range

**Table 2:** Diagnostic accuracy of 6-zone lung ultrasound

Lung pattern	LU/CT	CT +	CT -	Sensitivity (95% CI)	Specificity (95% CI)	LR + (95% CI)	LR—(95% CI)
Consolidation	LU +	118	12	0.76 (0.68 to 0.82)	0.92 (0.87 to 0.96)	9.8 (5.6 to 17.0)	0.26 (0.20 to 0.35)
	LU-	38	143				
Interstitial syndrome	LU +	48	24	0.60 (0.48 to 0.71)	0.69 (0.58 to 0.79)	2.0 (1.3 to 2.9)	0.58 (0.43 to 0.78)
	LU-	32	54				
Pneumothorax	LU +	10	4	0.59 (0.33 to 0.82)	0.97 (0.93 to 0.99)	20.7 (7.3 to 58.9)	0.42 (0.24 to 0.75)
	LU-	7	137				
Pleural effusion	LU +	94	10	0.85 (0.77 to 0.91)	0.77 (0.62 to 0.88)	3.7 (2.1 to 6.4)	0.19 (0.12 to 0.31)
	LU-	16	33				

Table depicting primary outcome, i.e., the diagnostic accuracy of a 6-zone lung ultrasound protocol. Two-by-two contingency Tables are displayed for consolidation, interstitial syndrome, pneumothorax and pleural effusion. A positive outcome for each outcome is denoted by a ‘ + ’, whereas a negative outcome is denoted by a ‘- ‘. Diagnostic accuracy parameters are estimated with their respective 95% confidence interval. LU: lung ultrasound; CT: computed tomography; CI: confidence interval; LR + : positive likelihood ratio; LR -: negative likelihood ratio

**Table 3:** Diagnostic accuracy of extended, 12-zone lung ultrasound protocol

Lung pattern	LU/CT	CT +	CT -	Sensitivity (95% CI)	Specificity (95% CI)	LR + (95% CI)	LR—(95% CI)
Consolidation	LU +	37	10	0.80 (0.66 to 0.91)	0.62 (0.41 to 0.80)	2.1 (1.3 to 3.5)	0.32 (0.16 to 0.62)
	LU-	9	16				
Interstitial syndrome	LU +	14	5	0.64 (0.41 to 0.83)	0.64 (0.35 to 0.87)	1.8 (0.8 to 3.9)	0.57 (0.29 to 1.1)
	LU-	8	9				
Pneumothorax	LU +	3	1	0.50 (0.12 to 0.88)	0.97 (0.83 to 1.0)	15.0 (1.9 to 120.9)	0.5 (0.2 to 1.1)
	LU-	3	29				
Pleural effusion	LU +	26	2	0.81 (0.64 to 0.93)	0.50 (0.07 to 0.93)	1.6 (0.6 to 4.4)	0.38 (0.11 to 1.27)
	LU-	6	2				

Table depicting secondary outcome, i.e., the diagnostic accuracy of a 12-zone lung ultrasound protocol. Two-by-two contingency Tables are displayed for consolidation, interstitial syndrome, pneumothorax and pleural effusion. A positive outcome for each outcome is denoted by a ‘ + ’, whereas a negative outcome is denoted by a ‘- ‘. Diagnostic accuracy parameters are estimated with their respective 95% confidence interval. BLUE: bedside lung ultrasound in emergency; LU: lung ultrasound; CT: computed tomography; CI: confidence interval; LR + : positive likelihood ratio; LR -: negative likelihood ratio

Prevalence of consolidations was 0.50 (156/311). Estimated sensitivity and specificity were 0.76 (95%CI: 0.68 to 0.82) and 0.92 (0.87 to 0.96), respectively. Prevalence of interstitial syndrome was 0.51 (80/158). Estimated sensitivity and specificity were 0.60 (95%CI: 0.48 to 0.71) and 0.69 (95%CI: 0.58 to 0.79), respectively. Prevalence of pneumothorax was 0.11 (17/158). Estimated sensitivity and specificity were 0.59 (95%CI: 0.33 to 0.82) and 0.97 (95%CI: 0.93 to 0.99), respectively. Out of the 17 pneumothoraces (hemithorax) three were clinically significant, i.e., replacement of the thoracic drain due to persistent pneumothorax in one case and surgical intervention due to persistent air leak in two cases. All three cases were correctly identified by lung ultrasound. Prevalence of pleural effusion was 0.71. Estimated sensitivity and specificity were 0.85 (95%CI: 0.77 to 0.91) and 0.77 (95%CI: 0.62 to 0.88), respectively.

**Fig. 2:**
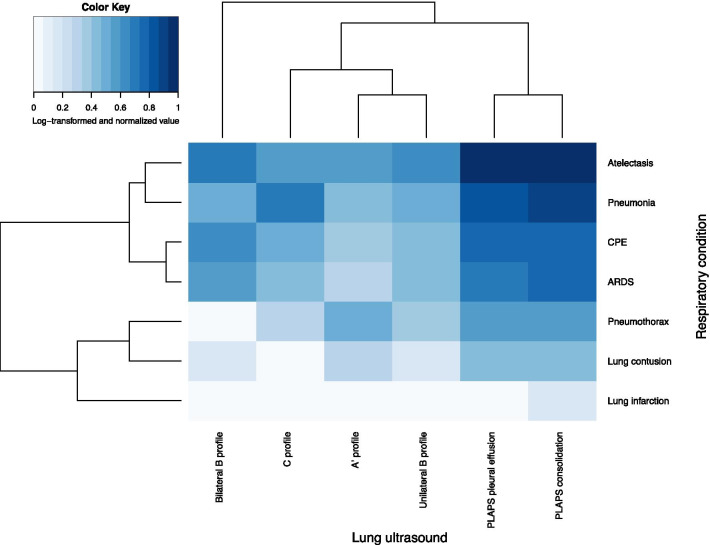
Heatmap showing correlation between respiratory conditions and lung ultrasound patterns. Dendrogram shows hierarchical clustering of respiratory conditions and lung ultrasound. For example, with respect to lung ultrasound pattern, CPE and ARDS appear to be similar conditions. To attenuate the effect of very prevalent conditions on coloring, such as atelectasis and posterior consolidations, the contingency data was first log-transformed and then normalized by the maximum cell value. ‘A’ profile’ represents an A-profile without lungsliding. ARDS: acute respiratory distress syndrome; CPE: cardiogenic pulmonary edema

In total there were 147 respiratory conditions in 79 patients. The majority (49.4%, 39/79) had two respiratory conditions. The most prevalent lung pathology was atelectasis, followed by pneumonia. The least prevalent lung pathology was lung infarction. Figure 2 shows a heatmap combining respiratory conditions and ultrasound patterns. Dendrograms show hierarchical clustering of respiratory conditions with respect to ultrasound patterns and vice versa.

## Discussion

The main findings of this prospective observational study are: 1. In a tertiary study population lung ultrasound is an accurate diagnostic modality to detect consolidation and pleural effusion, whereas the diagnostic accuracy to detect pneumothorax and interstitial syndrome is slightly lower. However, all clinically significant pneumothoraces were correctly identified by lung ultrasound. 2. The diagnostic accuracy did not seem to differ between the 6-zone BLUE-protocol and an extended 12-zone lung ultrasound protocol. 3. The majority of patients had at least two respiratory conditions, correlated to (partially) similar lung ultrasound patterns (Fig. 2). These findings are novel as, to our knowledge, this is the first study that investigated the full extent of lung ultrasound in ICU practice. Previous studies solely focused on one diagnosis (for example, ARDS, ILD or pneumonia) did not include anterior consolidations, or were inadequately blinded [8, 10, 12–15].

Sensitivity and specificity of lung ultrasound to detect consolidation are in line with previous research. However, in contrast to two studies with similar design, anterior consolidations were included as well [8, 13]. Anterior consolidations were defined as a C-profile or B-profile without lungsliding. Of note, a B-profile without lungsliding has been shown to be correlated to ARDS and pneumonia [7, 23]. Sensitivity and specificity for interstitial syndrome were lower than reported in the previous literature. We assume the reason to be twofold. First, sensitivity and specificity were affected by regarding a B-profile without lungsliding to be an anterior consolidation, whereas this might not always be the case. Second, ultrasound examinations were performed with a linear transducer, whereas recent literature suggests that B-lines are often better visualized using a microconvex or abdominal transducer [23, 24]. Sensitivity to detect pneumothorax was low, but specificity high. The number of areas of the lung covered by lung ultrasound examination offers an explanation; most false negatives were small apical or retrosternal pneumothoraces and these are easily missed by strictly following the BLUE-protocol. However, as our results show, an A-profile without lungsliding in ICU patients is highly indicative of pneumothorax. Sensitivity and specificity to detect pleural effusion were only slightly lower than previously reported [8, 13]. Again we believe this reason to be twofold. First, a small amount of pleural effusion in proximity of the spine is easily missed if patients are only examined in supine position, this reduces the sensitivity of lung ultrasound. Second, only a minimal amount of pleural effusion at one of the lung ultrasound points already renders the ultrasound examination positive, whereas in that case pleural effusion might not be visible on thoracic CT and, consequently, lowers the specificity.

Our study showed that an extended 12-zone lung ultrasound protocol is of little benefit in diagnosing lung pathology. Most diagnostic accuracy parameters were comparable to the 6-zone BLUE protocol (Table 2 and 3). This might be explained by the fact that almost all acute respiratory disorders involve a large portion of the subpleural space and are therefore easily accessible to ultrasound. In other words, if one or two of the anterior BLUE-points are involved in an acute respiratory disorder, the extra points scanned below the anterior axillary line are most likely involved as well. Moreover, if the PLAPS-point is involved, the extra point scanned more superiorly below the posterior axillary line is likely involved as well. Therefore, the 6-zone lung ultrasound protocol suffices and any additional points do not increase the diagnostic yield. Other studies have demonstrated the same results [25–27].

The presence of multiple respiratory conditions per patient in a tertiary ICU population is a common occurrence, but makes a diagnostic accuracy study difficult to perform. We addressed this issue by showing the correlation between lung ultrasound findings and respiratory conditions in a heatmap (Fig. 2). One should be careful to interpret the results in an absolute sense as the purpose is to provide the clinician with a tool to incorporate lung ultrasound in the diagnostic process. For example, if a C-profile is present, the differential diagnosis should contain pneumonia, but the possibility that a patient has an additional other diagnosis should not be disregarded. In our study, a patient who underwent an esophagectomy and gastric pull-up which was postoperatively complicated by an anastomotic dehiscence developed ARDS, a pneumothorax and compression atelectasis; all respiratory conditions could lead to acute respiratory failure. If one follows a deterministic protocol in any ICU setting, such as the one proposed by Mojoli et al.[19], one could easily miss other diagnoses if present. With the findings of the current study, we would like to emphasize the importance of using lung ultrasound as a diagnostic aid rather than as deterministic model with an exhaustive list of diagnoses. In the heatmap, respiratory conditions and lung ultrasound findings are clustered by a dendrogram. For example, with regard to ultrasound findings, ARDS and cardiogenic pulmonary edema are similar respiratory conditions. Both are characterized by bilateral B-lines, posterior consolidations and pleural effusion. In fact, the distinction between these conditions is notoriously difficult and is the subject of ongoing research (28, 29).

Due to its usefulness, the role of bedside ultrasound has been increasing in the intensive care setting. Not only lung ultrasound, but also transthoracic echocardiography have become standard care in many ICUs. Reasons for this surge in popularity are obvious: immediate bedside availability in deteriorating patients, absence of ionizing radiation and avoiding transportation to the radiology department. Despite these advantages, to deliver best patient care and avoid conflicts, well-deliberated agreements between ICU physicians and other medical specialties are necessary, especially in cardiology and radiology. For example, in case of ultrasound of the heart the intensivist should be able to qualitatively assess the systolic function of the heart or exclude important conditions such as cardiac tamponade, whereas quantitative assessment of systolic function and other more advanced measurements remain mostly reserved for echocardiography performed by the cardiologist. Emphasizing the importance of careful deliberation, in case of more advanced trained ICU physicians – for example with an European Diploma in Advanced Critical Care Echocardiography (EDEC) – departments can decide to change these agreements.

This study has several limitations. First, it was a single-center observational study conducted in a tertiary ICU population; a substantial part of the study population was immunocompromised or underwent complicated surgical procedures. Second, lung ultrasound examinations were performed by multiple ultrasound operators with different levels of experience. More experienced operators are more likely to detect subtle lung ultrasound abnormalities and, thus, are better able to correctly classify each examination. However, all operators followed a basic ICU point-of-care ultrasound course and were able to perform examinations. With this in mind, our study results are indeed better generalizable than results of a study with just a single experienced lung ultrasound operator. Moreover, ultrasound examinations are inherently dynamic and interpretation is operator dependent. Because of the operator dependency, it requires all medical staff to be trained in performing ultrasound examinations. In contrast, CT is often performed by a technician and images can then be remotely reviewed by a single radiologist. However, in an acute situation it is important to rapidly obtain a diagnosis and, in our opinion, ICU physicians should be able to perform basic ultrasound examinations. Major strengths of our study are that this is, to our knowledge, the first study that included anterior consolidations and pneumothorax, is adequately blinded and does not exclude patients with multiple diagnoses. Due to the fact that this study allowed for the possibility of multiple respiratory conditions, these results are very representative of and applicable to routine ICU practice. Most importantly, this is also one of the largest studies regarding the diagnostic accuracy of lung ultrasound performed on a tertiary ICU population.

## Conclusions

In conclusion, lung ultrasound is an adequate diagnostic modality in a tertiary ICU population to detect consolidation, clinically significant pneumothorax and pleural effusion and is less suitable to detect interstitial syndrome. A 12-zone lung ultrasound protocol shows no benefits over the 6-zone lung ultrasound protocol. Moreover, one should be careful not to interpret lung ultrasound findings in deterministic fashion as most patients have multiple respiratory conditions associated with (partially) similar lung ultrasound patterns.

## Supplementary Information

**Additional file 1.** Standards for Reporting Diagnostic accuracy studies (STARD) checklist.

## Data Availability

Original data and analyses are available from the corresponding author on reasonable request.

## Abbreviations

ARDS: Acute respiratory distress syndrome
BLUE: Bedside lung ultrasound in emergency
CT: Computed tomography
HU: Houndsfield units
ICU: Intensive care unit
ILD: Interstitial lung disease
IQR: Interquartile range
PLAPS: Posterolateral alveolar and/or pleural syndrome
SD: Standard deviation
